# DNA methylation in demyelinated multiple sclerosis hippocampus

**DOI:** 10.1038/s41598-017-08623-5

**Published:** 2017-08-18

**Authors:** Anthony M. Chomyk, Christina Volsko, Ajai Tripathi, Sadie A. Deckard, Bruce D. Trapp, Robert J. Fox, Ranjan Dutta

**Affiliations:** 1 Department of Neurosciences, Cleveland Clinic, Cleveland, OH 44195 USA; 2 Mellen Center for MS Research, Cleveland Clinic, Cleveland, OH 44195 USA

## Abstract

Multiple Sclerosis (MS) is an immune-mediated demyelinating disease of the human central nervous system (CNS). Memory impairments and hippocampal demyelination are common features in MS patients. Our previous data have shown that demyelination alters neuronal gene expression in the hippocampus. DNA methylation is a common epigenetic modifier of gene expression. In this study, we investigated whether DNA methylation is altered in MS hippocampus following demyelination. Our results show that mRNA levels of DNA methyltransferase were increased in demyelinated MS hippocampus, while de-methylation enzymes were decreased. Comparative methylation profiling identify hypo-methylation within upstream sequences of 6 genes and hyper-methylation of 10 genes in demyelinated MS hippocampus. Genes identified in the current study were also validated in an independent microarray dataset generated from MS hippocampus. Independent validation using RT-PCR revealed that DNA methylation inversely correlated with mRNA levels of the candidate genes. Queries across cell-specific databases revealed that a majority of the candidate genes are expressed by astrocytes and neurons in mouse and human CNS. Taken together, our results expands the list of genes previously identified in MS hippocampus and establish DNA methylation as a mechanism of altered gene expression in MS hippocampus.

## Introduction

Multiple sclerosis (MS) is an inflammatory, demyelinating, and neurodegenerative disease of the central nervous system (CNS) that affects more than two million people worldwide^1, 2^. Among the spectrum of cognitive impairments, memory dysfunction is most common among MS patients^3, 4^. Hippocampal demyelination is extensive in individuals with MS and modulates expression of neuronal genes involved in synaptic plasticity and memory function^5, 6^.

Large-scale genome-wide association studies (GWAS) have identified MS susceptibility loci, including human leukocyte antigen loci and other immune-function related genes^7–10^; however, their functional significance related to MS pathogenesis is still unknown. The relatively low concordance rate of single-nucleotide polymorphisms in monozygotic twins^11^, the presence of a strong gender bias, and the influence of migration on disease onset collectively suggest that the pathogenesis of MS likely results from a combination of both genetic and epigenetic factors^12, 13^. Epigenetic modifications, including DNA methylation, histone modification, chromatin remodeling, and noncoding RNA regulation have been reported to regulate gene expression and to participate in the etiology of MS^14, 15^.

DNA methylation occurs in special genomic regions called CpG islands, which contain greater than 50% cytosine and guanine nucleotides. It plays a major role in aberrant expression of genes that are important in several neurological diseases^16, 17^ as well as in memory formation and maintenance^18, 19^. We previously compared and identified several genes and microRNAs in MS hippocampus that correlate with synaptic changes, memory dysfunction, and hippocampal demyelination^5, 20^.

In this study, we investigated additional epigenetic mechanisms that alter gene expression in MS hippocampus. We found significant increases in mRNA levels of key DNA methyltransferase enzymes (DNMTs). Interestingly, the mRNA levels of the three DNA de-methylation enzymes (ten-eleven translocation methylcytosine dioxygenase 1–3; TET1-TET3), which catalyze hydroxy-methylation as well as the total level of hydroxy-methylated residues, were significantly decreased in MS demyelinated hippocampus. Several differentially methylated positions (DMPs) were also identified by comparing MS myelinated to demyelinated hippocampus. The methylation status of DMPs inversely correlated with mRNA levels of target genes that have been associated with neuronal survival and memory function. As methylation patterns in different cell types may contribute to overall methylation patterns^21^, human and mouse cell-specific databases indicated that a majority of the target genes were localized to neurons and astrocytes. Collectively, our results provide evidence that DNA methylation could play a major role in controlling gene expression in MS hippocampus.

## Results

### Demyelination correlates with changes in DNA methylation and de-methylation enzymes in MS hippocampus

**Figure 1:**
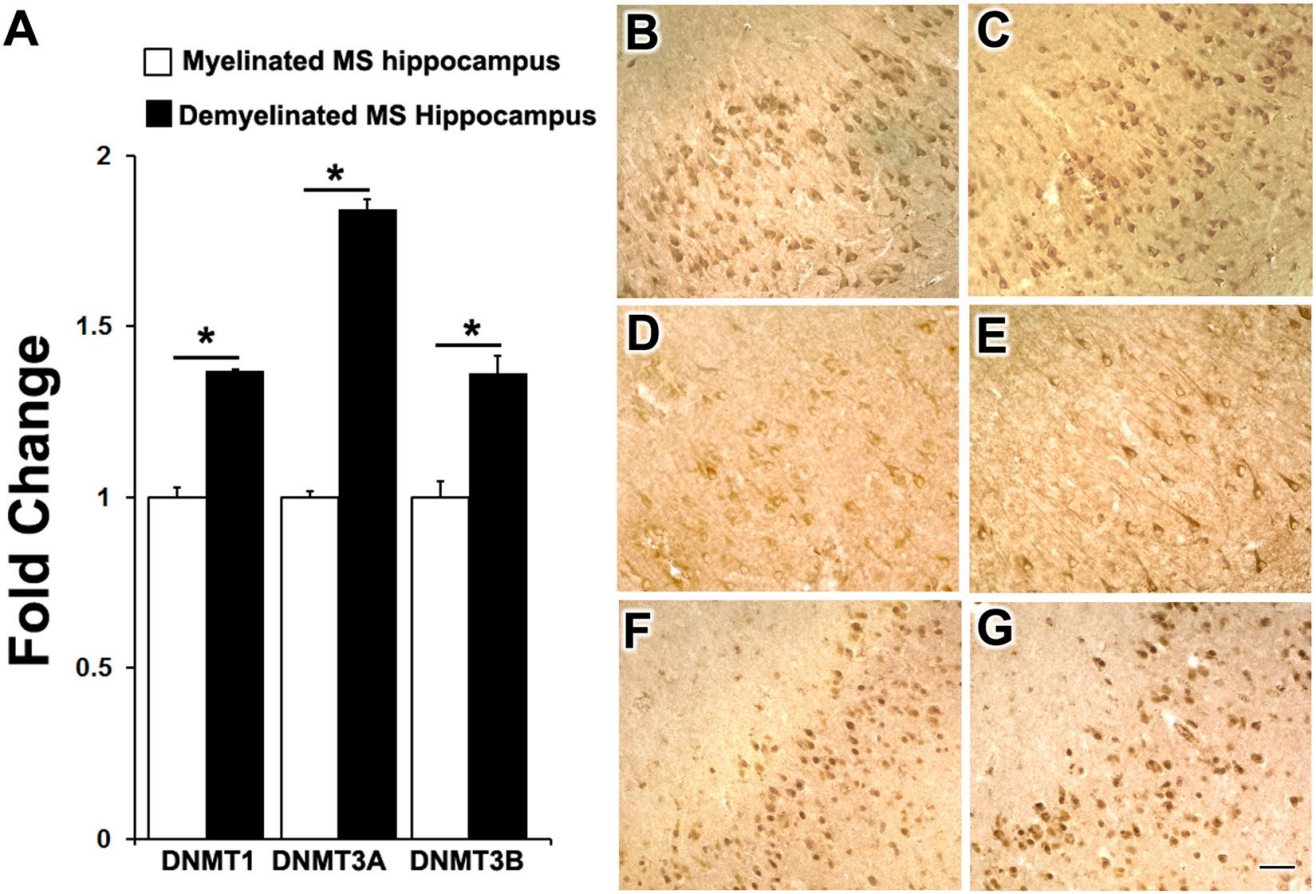
DNA methyltransferase (DNMT) expression in hippocampi from multiple sclerosis (MS) brains. RT-PCR analysis shows significant increases in mRNA levels of DNMT 1, DNMT3A, and DNMT3B in demyelinated hippocampus (n = 4) compared to myelinated hippocampus (n = 4) (**A**). Immunohistochemistry showing cellular expression of DNMT1, DNMT3A, and DNMT3B in myelinated (**B**,**D**,**F**) and demyelinated hippocampus (**C**,**E**,**G**), with predominant expression in hippocampal neurons. Scale Bars: B–G: 30 μm; Error bars indicate + S.E.M.; *p < 0.05.

In order to study DNA methylation, we measured levels of DNMT enzymes responsible for inserting and maintaining DNA methylation (DNMT1, DNMT3A, and DNMT3B) in myelinated and demyelinated MS hippocampi. Demyelination led to significant increases in mRNA levels of all three DNMTs in MS hippocampus (Fig. 1A). Immunohistochemical analysis showed that DNMT1 (Fig. 1B,C), DNMT3A (Fig. 1D,E) and DNMT3B (Fig. 1F,G) were primarily associated with hippocampal neurons in MS myelinated (Fig. 1B,D,F) and MS demyelinated (Fig. 1C,E,G) hippocampus.

**Figure 2:**
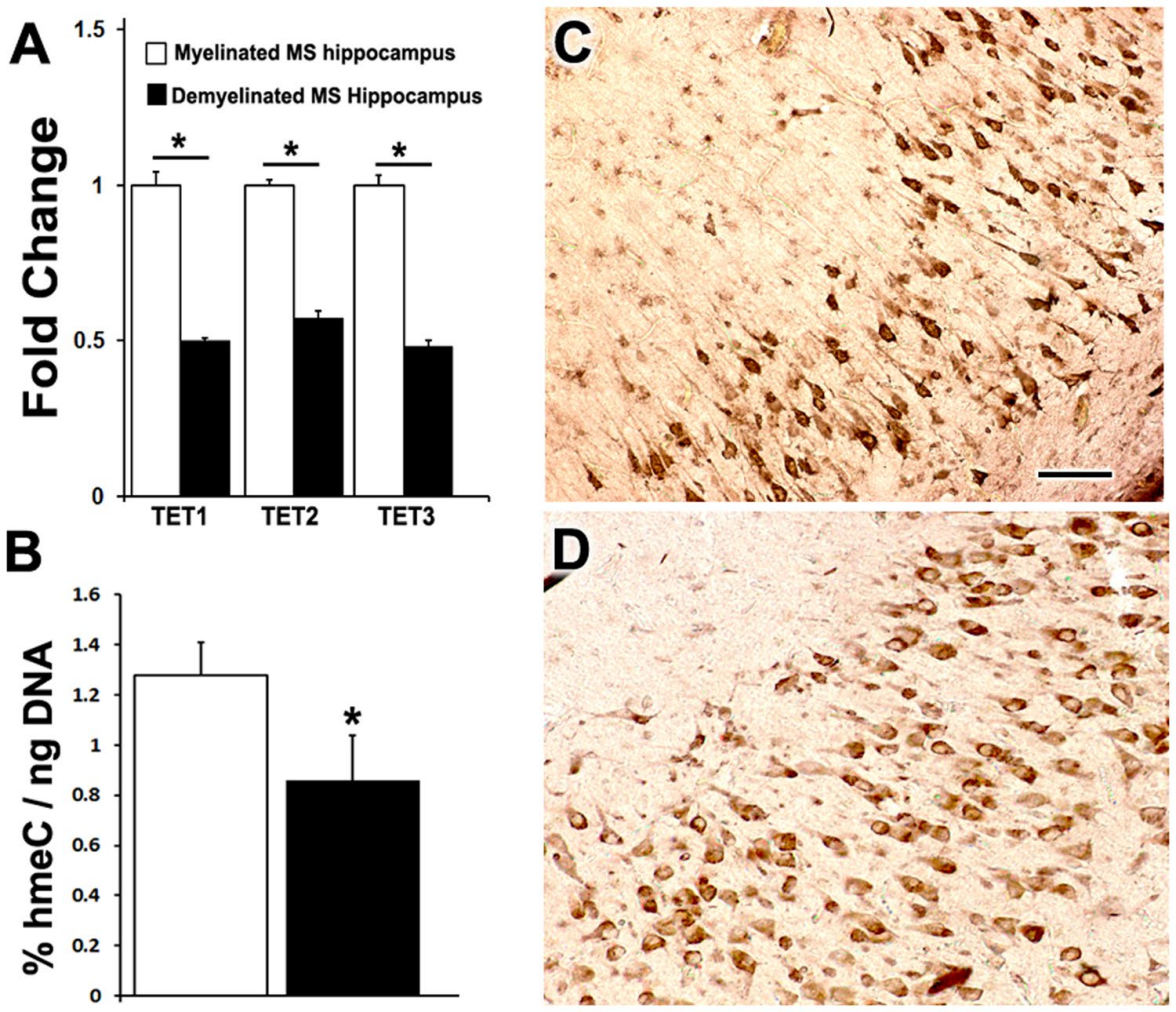
Demyelination in MS brains leads to lower expression of TET enzyme mRNA and hydroxymethyl residues. RT-PCR analysis shows significant decreases in mRNA levels of TET 1, TET 2, and TET 3 in demyelinated hippocampus (n = 4) compared to myelinated hippocampus (n = 4) (**A**). Compared to myelinated hippocampus (n = 8), demyelination (n = 7) led to a significant decrease in levels of hydroxymethyl content (hmC) (**B**). Immunohistochemistry analysis using a 5hmC antibody shows neurons as the major cell type with 5 hmC expression in myelinated (**C**) and demyelinated hippocampus (**D**). Scale Bars: C–D: 30 μm; Error bars indicate + S.E.M.; *p < 0.05.

In concert with DNMT enzymes inserting and maintaining methylation patterns, the TET family of de-methylation enzymes oxidizes methylcytosine (5 mC) to hydroxymethylcytosine (5 hmC) to remove methylation^22^. Three TET proteins (TET 1–3) have been identified in humans^23^, which all convert 5 mC to 5 hmC. Given the relationship between methylation and TET enzymes, we also quantified mRNA levels of TET1–3 in MS hippocampus (Fig. 2A). The results showed a significant decrease in mRNA levels of all three TET genes in demyelinated hippocampi from individuals with MS. In order to test whether the lower mRNA levels of TET enzymes also correlated with levels of 5hmC residues, we measured the global levels of 5hmC within DNA isolated from MS myelinated and demyelinated hippocampi. The results showed that there was a significant decrease in the overall level of 5hmC within demyelinated hippocampus that correlated with the decreased mRNA levels of TET1–3 (Fig. 2B). We then conducted histochemical analysis to determine the cellular expression of 5hmC and we determined that neurons were the major cell type containing 5hmC in MS myelinated (Fig. 2C) and demyelinated hippocampus (Fig. 2D).

### Loss of myelin correlates with DNA methylation changes within transcription start sites

**Figure 3:**
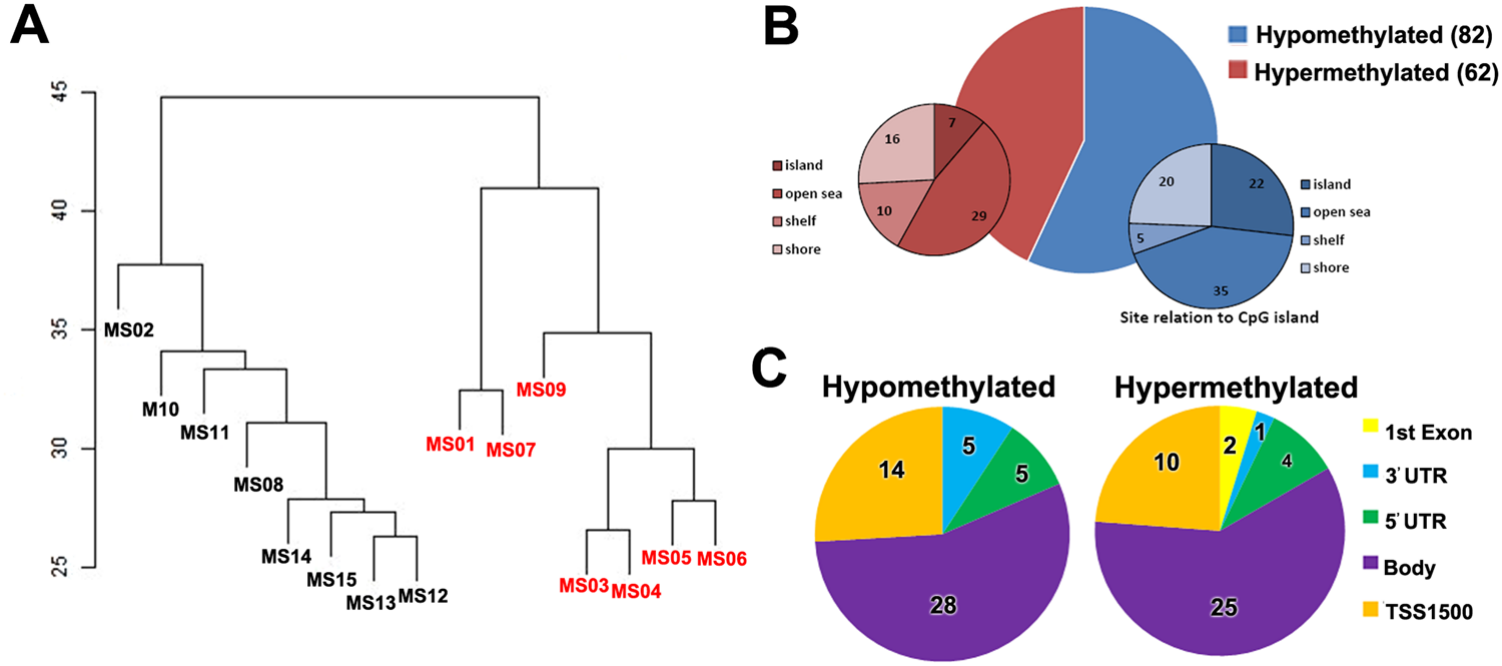
Global methylation profiles are different between myelinated and demyelinated MS hippocampus. Global DNA methylation was assessed using the Illumina 450 K array. Resultant clustering analysis shows that the MS demyelinated samples (shown in red) are separated from MS myelinated samples (**A**). Compared to myelinated MS hippocampus, 144 DMPs (62 hyper-methylated, 82 hypo-methylated) were identified in MS demyelinated hippocampus (**B**). Localization of these DMPs based on CpG islands, flanking CpG Islands (CGIs) (shores and shelves; 2–4 kb from CGIs), and open sea (non-related to CpGs) are shown (**C**). DMPs mapped onto genomic features (‘1^st^ exon’, 3′ and 5′ UTRs, ‘body’ of the gene, within 1500 bp of the transcription start site (TSS) of well characterized genes (UCSD genome browser hg19) show differences between hypo- and hyper-methylated targets.

Imprinted gene expression is regulated by epigenetic mechanisms, particularly DNA methylation at DMPs. We did not detect any significant changes in overall 5mC levels in DNA from myelinated or demyelinated MS hippocampus (data not shown). We therefore used a more sensitive global array-based approach to identify DMPs by comparing myelinated (n = 8) and demyelinated (n = 7) MS hippocampus (Fig. 3A) using an Infinium HumanMethylation 450 K array (Illumina Inc, USA). Patient details are in Table 1. The 450 k array contains 485,512 probes covering 99% of RefSeq genes. The probes interrogate 19,755 unique CpG islands with additional coverage in shore regions as well as 3091 probes at non-CpG sites^24^. Resultant methylation assays and global clustering showed that the methylation pattern could reliably differentiate between myelinated and demyelinated MS hippocampus (Fig. 3A). Demyelination resulted in identification of 144 DMPs (62 hypermethylated, 82 hypomethylated) within the assessed CpG sites (adjusted *p* < 0.05, delta β 20%; Fig. 3B). Localization of these DMPs in the context of genomic location was classified into different categories (Fig. 3C): CpG islands, flanking CpG Islands (CGIs) (shores and shelves; 2–4 kb from CGIs), and open sea (not related to CpGs). We found 29 out of 62 (46.7%) DMPs detected within the CpG islands had increased methylation, whereas 26.8% (22 out of 82) of the DMPs within the CpG islands showed decreased methylation. We next asked whether these DMPs fall within the genomic location of well-characterized genes (UCSD genome browser hg119). The DMPs were mapped to either the ‘1^st^ exon’, 3′ and 5′ UTRs, or the ‘body’ (non-exonic) of the gene, or within 1500 bp of the transcription start site (TSS). The results showed that 92 out of 144 altered DMPs (65%) were mapped to 75 annotated genes (UCSD genome browser hg119). Several of the identified genes had multiple DMPs associated with their sequences (Table 2). To further validate our findings, we compared these identified genes with results obtained from an independent gene microarray database using MS hippocampus^5^. The results showed that a significant number of genes (43 out of 75) identified in the current study were also altered at the mRNA expression level in the previous study (gene symbols in bold letters, Tables 2 and 3).

**Table 1:** Demographics of MS patients.

	Type of MS	Hippocampal Myelin Status	Disease Duration (yrs)	Age(yrs)/Sex	EDSS	PMI (hrs)
MS 01	SPMS	Demyelinated	38	59/F	9.0	5
MS 02	SPMS	Myelinated	10	65/F	3.0	9
MS 03	SPMS	Demyelinated	45	70/F	9.5	4
MS 04	RRMS	Demyelinated	10	49/M	9.0	18
MS 05	SPMS	Demyelinated	14	62/M	8.0	5
MS 06	SPMS	Demyelinated	29	49/F	9.0	4
MS 07	SPMS	Demyelinated	9	46/F	9.0	7
MS 08	PPMS	Myelinated	13	64/M	8.0	4
MS 09	SPMS	Demyelinated	46	73/F	6.5	7
MS 10	SPMS	Myelinated	14	55/M	7.5	7
MS 11	PPMS	Myelinated	39	61/F	8.0	12
MS 12	SPMS	Myelinated	30	75/F	8.0	8
MS 13	SPMS	Myelinated	27	71/F	9.5	5
MS 14	SPMS	Myelinated	16	61/F	6.0	8
MS 15	SPMS	Myelinated	37	63/M	7.5	7
		Average	25.1	61.3	7.8	7.3

SPMS: Secondary Progressive Multiple Sclerosis; PPMS: Primary Progressive Multiple Sclerosis; RRMS: Relapsing Remitting Multiple Sclerosis. EDSS: Expanded Disability Status Scale; PMI: Postmortem Interval.

**Table 2:** Cellular identity of hypo- and hyper-methylated CpG target genes identified in MS hippocampus following demyelination.

**Hypomethylated**			**Mouse**				**Human**			
**3'UTR**	**probeID**	**Gene Name**	**A**	**N**	**O**	**M**	**A**	**N**	**O**	**M**
**MLLT4**	cg06738063	**Myeloid/Lymphoid or Mixed-Lineage Leukemia**	X	X			X	X		
**PPIF**	cg26584456	**Peptidylprolyl Isomerase F**		X						X
SCRT2	cg24595510	Scratch Family Zinc Finger 2		X						
	cg07482223									
**SNRNP40**	cg22802014	**Small Nuclear Ribonucleoprotein 40kDA**	X	X		X		X	X	X
**5'UTR**	**probeID**	**Gene Name**								
ISLR2	cg19470379	Immunoglobulin Containing Leucine-Rich Repeat 2		X						
	cg25666233									
**MEF2A**	cg00062736	**Myocyte Enhancer Factor 2A**		X		X		X		X
**PMEPA1**	cg26681770	Prostate Transmembrane Protein, Androgen Induced 1				X		X		X
	cg04628369									
**Body**	**probeID**	**Gene Name**								
**ABCA4**	cg04350215	**ATP-Binding Cassette, Subfamily A, Member 4**					X			
**ADAMTS12**	cg10594543	**ADAM Metallopeptidase TS12**	X							
**AHRR**	cg23576855	**Aryl-Hydrocarbon Receptor Repressor**	X	X						X
BEST3	cg03196364	Bestrophin 3							X	
**CASP7**	cg01128042	**Caspase 7, Apoptosis-Related Cysteine Peptidase**	X			X	X			
CCL4L2	cg04850148	Chemokine (C-C Motif) Ligand 4-Like 2								X
**CPXM2**	cg01512466	**Carboxypeptidase X (M14 Family), Member 2**	X							
FBXW8	cg02017074	F-Box and WD Repeat Domain Containing 8	X	X	X	X	X			
**HLA-B**	cg23427945	**Human Leukocyte Antigen, Class I, B**								X
LOC145845	cg00216138	Uncharacterized LOC 145845								
	cg13020870									
	cg25718467									
	cg24956533									
	cg21375869									
**MEIS1**	cg06833110	**Meis Homeobox 1**	X				X		X	
**MGMT**	cg07638938	**O-6-Methylguanine-DNA Methyltransferase**				X	X	X		
**MYO7A**	cg17355865	**Myosin VIIA**				X				X
NXN	cg19669385	Nucleordeoxin		X						
	cg08190450									
**PKP2**	cg03762760	**Plakophilin 2**	X					X		
**PQLC1**	cg20218571	**PQ Loop Repeat Containing 1**				X				X
**PSD3**	cg10695549	**Pleckstrin and Sec7 Domain Containing 3**	X	X				X		
SCN4B	cg22251955	Sodium Channel, Voltage Gated, Type IV B		X			X	X		
**SDK2**	cg05787106	**Sidekick Cell Adhesion Molecule 2**	X	X			X	X		
**SMYD3**	cg06999043	**SET and MYND Domain Containing 3**		X		X	X	X		
**TGFBI**	cg17386240	**Transforming Growth Factor, Beta-Induced**				X				X
**TMEM165**	cg00532122	**Transmembrane Protein 165**	X						X	
**Hypomethylated**			**Mouse**				**Human**			
**3′UTR**	**probeID**	**Gene Name**	**A**	**N**	**O**	**M**	**A**	**N**	**O**	**M**
**PON1**	cg09416203	**Paraoxonase 1**					X			
**5′UTR**	**probeID**	**Gene Name**								
**HDLBP**	cg17240976	**High Density Lipoprotein Binding Protein**	X			X	X	X		
**MKKS**	cg08331829	**McKusick-Kaufman Syndrome**	X		X			X		
**TRIM26**	cg10985055	**Tripartite Motif Containing 26**				X		X		
**TRPS1**	cg04613734	**Trichorhinophalangeal Syndrome 1**	X				X			
**1** ^**st**^ **Exon**	**probeID**	**Gene Name**								
KRTAP27-1	cg05809586	Keratin Associated Protein 27-1								
MGP	cg06601891	Matrix Gla Protein						X		
**Body**	**probeID**	**Gene Name**								
**AJAP1**	cg00345083	**Adherens Junctions Associated Protein 1**		X				X		
**C1orf106**	cg10092377	**Chromosome 1 open reading frame 106**							X	
C2orf62	cg13215060	Ciliogenesis Associated TTC17 Interacting Protein								
**DSE**	cg24407607	**Dermatan Sulfate Epimerase**		X		X				X
EIF2C2	cg14708514	Eukaryotic Translation Initiation Factor 2C, S 2	X	X			X			X
GATA5	cg00286102	GATA Binding Protein 5								
**HLA-B**	cg19493134	**Human Leukocyte Antigen, Class I, B**								X
**IGSF9B**	cg25790212	**Immunoglobulin Superfamily, Member 9B**		X						
INSC	cg24136292	Inscuteable Homolog (Drosophila)			X					
**KIAA1026**	cg25307521	**Kazrin, Periplakin Interacting Protein**								
**KIF25**	cg24246628	**Kinesin Family Member 25**								
	cg14316629									
LOC100292680	cg24730756	Uncharacterized LOC100292680								
**NFASC**	cg23564471	**Neurofascin**			X			X	X	
**RASA3**	cg27086157	**RAS P21 Protein Activator 3**	X			X				X
SDK1	cg24441899	Sidekick Cell Adhesion Molecule 1				X				X
SHISA2	cg05918715	Shisa Family Member 2		X					X	
**SOLH**	cg26722972	**Calpain 15 or Small Optic Lobes Homolog**	X	X						
**SORBS2**	cg09120722	**Sorbin and SH3 Domain Containing 2**		X				X		
**TAGLN3**	cg08522473	**Transgelin 3**	X	X				X		
**TBX5**	cg06725552	T-Box 5								
	cg11841394									
**TM9SF1**	cg02898977	**Transmembrane 9 Superfamily Member 1**	X			X	X			
TOP1MT	cg00033213	Topoisomerase (DNA)I, Mitochondrial			X					
ZSCAN1	cg27002247	Zinc Finger and SCAN Domain Containing 1								

Identified genes were mapped to astrocytes (A), neurons (N), oligodendrocytes (O), or microglia/macrophage lineage (M) cells in mouse and human CNS cell-specific database^25, 26^. To increase confidence in cellular identity, matching genes showing expression levels above the 50^th^ percentile were selected. Genes significantly altered in a comparison of MS hippocampus using previously published microarray-based analsis^5^ are shown in bold.

**Table 3:** Cellular Identity of hypo- and hyper-methylated CpGs near TSS in human and mouse.

Hypomethylated			Mouse				Human			
TSS1500	probeID	Gene Name	A	N	O	M	A	N	O	M
*AKNA*	*cg13910813*	*AT-Hook Transcription Factor*				X				X
*EBPL*	*cg04663916*	*Emopamil Binding Protein-like*		X	X	X	X	X		
FLJ42709	cg15444648	Uncharacterized LOC441094					X	X		
	cg05977002									
	cg16600634									
	cg07546139									
***HERC6***	*cg08684066*	***HECT Domain Containing E3 UBL Member 6***	X					X		
OR52M1	cg17040924	Olfactory Receptor, Family 52, Subfamily M1								
***SFRP1***	*cg03575666*	***Secreted Frizzled-Related Protein 1***	X	X				X	X	
	*cg14824386*									
	*cg23359714*									
	*cg01074584*									
	*cg00930833*									
	*cg06166767*									
**Hypermethylated**			**Mouse**				**Human**			
**TSS1500**	**probeID**	**Gene Name**	**A**	**N**	**O**	**M**	**A**	**N**	**O**	**M**
C22orf43	cg11466708	Aspartate-Rich 1 or Chromosome 22 ORF43								
LOC285830	cg12035144	Uncharacterized LOC 288530								
NAPEPLD	cg11692070	N-Acyl Phosphatidylethanolamine D			X		X	X	X	
***NHLH2***	*cg22427279*	***Nascent Helix Loop Helix 2***		X						
***PLCH1***	*cg02344477*	***Phospholipase C, Eta 1***	X				X	X		
SERPINA9	cg13251750	Serpin Peptidase Inhibitor, Member 9								
SLFN13	cg00364956	Schlafen Family Member 13								
*TMEM132B*	*cg01012836*	*Transmembrane Protein 132B*	X		X		X	X		
**TTLL3**	cg06870118	**Tubulin Tyrosine Ligase-Like 3**	X	X	X	X	X			
*WDR81*	*cg03854564*	*WD Repeat Domain*				X				

**Figure 4:**
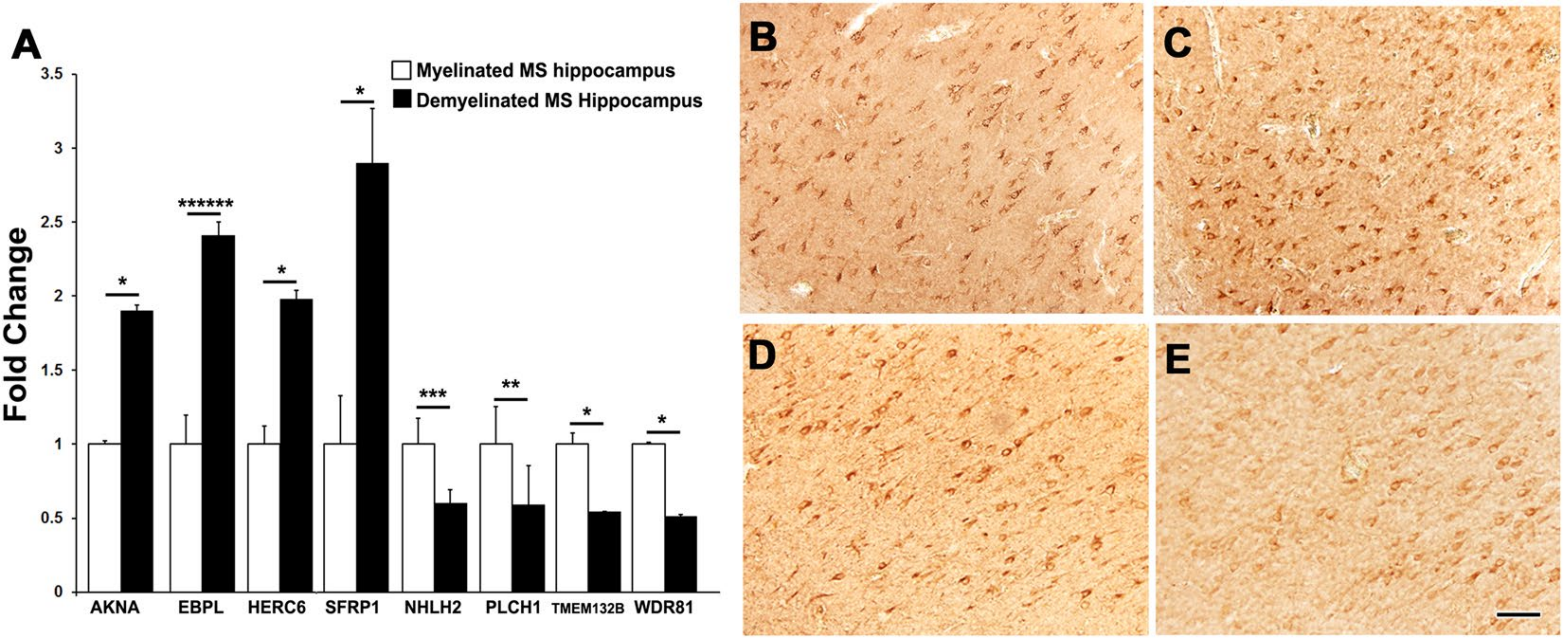
Inverse correlation between DMP and mRNA levels of target genes. RT-PCR analysis shows significant increases in mRNA levels of AT-hook transcription factor (AKNA), Emopamil Binding Protein Like (EBPL), HECT domain and RCC1-like domain containing protein 6 (HERC6), and Secreted frizzled related protein 1 (SFRP1) in demyelinated MS hippocampus (n = 4) compared to myelinated MS hippocampus (n = 4). Hyper-methylation within Nescient helix-loop-helix 2 (NHLH2), Phospholipase C eta 1 (PLCH1), Transmembrane protein 132B (TMEM132B), and WD repeat domain 81 (WDR81) following demyelination led to significant decreases in mRNA levels. Immunohistochemistry showing cellular expression of SFRP1 and PLCH1 in myelinated (**B**,**D**) and demyelinated hippocampus (**C**,**E**), with predominant expression in hippocampal neurons. Scale Bars: B–E: 30 μm; Error bars indicate + S.E.M.; * p < 0.05, **p < 0.005, ***p < 0.0005, *****p < 0.000005.

Genes where altered methylation was detected within 1500 bp were matched to corresponding cell types. Genes significantly altered in a comparison of MS hippocampus using previously published microarray-based analysis^5^ are shown in bold. mRNA levels of genes measured using RT-PCR and presented in Figure 4 are italicized.

### Identification of cell types expressing transcripts containing DMPs in mouse CNS

After we identified altered DMPs and their target genes in MS hippocampus, we compared the cellular expression of these target genes in mouse CNS using published mouse and human RNA-seq databases^25, 26^. This comparison revealed that DMPs are associated with genes expressed by the four major cell types (astrocytes, neurons, oligodendrocytes, and microglia) in mouse and human CNS (Table 2). Among identified hypomethylated targets (within UTR, gene body, and exon), 41% were specific to astrocytes, whereas 44% (13 out of 29) of the target genes were expressed by neurons in mouse CNS. The results also showed that 34% of the target genes were expressed by microglia/macrophage lineage cells in mouse (Table 2). Interestingly, hypomethylation identified 1 gene in mouse oligodendrocytes (F Box and WD repeat domain 8) and 4 in human oligodendrocytes (Small Nuclear Ribonucleoprotein 40 kDA; Bestrophin 3; Meis Homeobox 1; and Transmembrane Protein 165). None of the genes were expressed by both mouse and human oligodendrocytes. Among the hypermethylated transcripts, (within UTR, gene body, and exon), 26% of the genes were expressed by astrocytes and neurons (8 out of 30), while 20% were also expressed by microglia/macrophage cells in mouse brain (Table 2). Four hypermethylated genes were oligodendrocyte-specific in mouse brain (McKusick-Kaufmna Syndrome; Inscuteable Homolog (Drosophila); Neurofascin; and Topoisomerase (DNA) I, Mitochondrial) and three (Chromosome 1 open reading frame 106; Neurofascin; and Shisa Family Member 2) were localized to human oligodendrocytes. Cellular analysis revealed that the hypermethylated, oligodendrocyte-specific Neurofascin 155, which is involved in maintenance of axoglial junctions^27^, is expressed by both mouse and human oligodendrocytes.

As the presence of DMPs within the promoter and TSS has the greatest possibility of affecting mRNA expression^14^, we identified the genes where a DMP was identified within 1500 bp of the TSS (Table 2). Our results showed that there were 14 DMPs (targeting 6 genes) that showed decreased methylation, while 10 DMPs (identified with 10 genes) showed increased methylation. Due to the presence of DMPs within 1500 bp of the TSS, we determined the cellular identification of the targets in both mouse and human RNA databases^25, 26^. The comparative results identified several genes that are differentially expressed by mouse and human CNS cells (Table 3). As brain tissue is a heterogeneous mixture of several cell types, these results not only provide clues as to the cellular specificity of each of the target genes, but they also provide options for further genetic manipulations in mouse models.

### DNA methylation changes within transcription start sites of genes inversely correlate with mRNA levels of target genes

DNA methylation at the proximity of the TSS is generally considered to be a potent epigenetic modification that prohibits transcription factor (TF) recruitment, resulting in transcription suppression^28, 29^. We therefore validated whether the presence of DMPs within 1500bp of the TSS affects mRNA levels in 4 hyper-methylated and 4 hypo-methylated target genes. The results showed (Fig. 4) that hypomethylation within the TSS1500 of AT-hook transcription factor (AKNA), Emopamil Binding Protein Like (EBPL), HECT domain, RCC1-like domain containing protein 6 (HERC6), and Secreted frizzled related protein 1 (SFRP1) led to significant increases in mRNA levels of these genes following demyelination. In contrast, hyper-methylation identified within the TSS1500 of Nescient helix-loop-helix 2 (NHLH2), Phospholipase C eta 1 (PLCH1), Transmembrane protein 132B (TMEM132B), and WD repeat domain 81 (WDR81) correlated with the decrease in mRNA levels of these genes following demyelination in MS hippocampus. Immunohistochemical analysis to confirm cellular localization, showed that SFRP1 (Fig. 4B,C), and PLCH1 (Fig. 4D,E) were primarily associated with hippocampal neurons in MS myelinated (Fig. 4B,D) and MS demyelinated (Fig. 4C,E) hippocampus.

## Discussion

Epigenetic targets of gene regulation provide interesting targets for therapeutics because they are modifiable. The present study identifies DNA methylation as a correlate of demyelination and gene expression in MS hippocampus. Demyelination coincides with increases in mRNA levels of DNA methylation enzymes, with concomitant decreases in levels of DNA demethylation enzymes. Global methylation analysis identified 144 DMPs in MS hippocampus targeting several genes expressed by the major cell types in the human brain. As human tissue is not amenable to experimental manipulations, we also performed a comparative database search to determine the cellular specificity of the candidate genes in mouse brain. These data were further screened to identify the 25 DMPs localized within 1500 bp of TSS of 16 genes. Independent validation revealed that mRNA levels of the identified genes inversely correlated with DNA methylation status. Previous studies have shown that hippocampal demyelination is common in MS patients, which leads to loss of synaptic density and can influence expression of synaptic and neuronal genes as well as regulate the expression of neuronal miRNAs^5, 20^. In this study, we identified candidate genes that are altered by methylation changes following demyelination in MS hippocampus and that may play a role in altering synaptic plasticity, memory performance, and neuronal survival in MS brains.

The presence/loss of methylation within the TSS alters mRNA levels of the target genes. We identified 6 genes where demyelination led to decreased methylation. Among these, mRNA levels of AKNA were significantly increased following demyelination. AKNA is a major regulator of CD40 and CD40 ligand^30^ and is reported in RNA seq databases to be expressed by microglia/macrophage cells (Table 3) in both mouse and human. Increases in microglial CD40 expression and interactions with its ligand CD40L have been shown to induce/stimulate the expression of tumor necrosis factor-alpha (TNF-α) and to initiate neuronal death^31^. Following demyelination, we detected a significant increase in methylation within the TSS of WDR81, a gene involved in neuronal survival^32^. Increased levels of AKNA in conjunction with decreased WDR81 following demyelination could therefore lead to increased levels of TNF-α and neuronal injury. In addition, we also identified hypomethylation and a significant increase in mRNA levels of SFRP1 following demyelination. SFRP1 is an inhibitor of the WNT signaling proteins, which regulate learning and memory as well as synaptic plasticity at central synapses^33, 34^. Our comparative methylation analysis also identified hypermethylation (near the TSS) and decreased mRNA levels of NHLH2 and PLCH1 (Fig. 4). NHLH2 is a positive regulator of melanocortin receptors and modulates memory and learning^35, 36^, while PLCH1 loss is generally associated with impaired working memory^37^. Differential methylation and concomitant increased levels of SFRP1 as well as decreased NHLH2 and PLCH1 could therefore lead to the decreased synaptic density and memory performance that have been previously reported following demyelination^5, 20^.

The ongoing discovery of epigenetic factors underlying pathogenesis in MS is substantial^14^. These factors are relatively easily modifiable, in many cases utilizing readily available pharmacological agents targeting epigenetic modifiers. The study of DNA methylation in MS pathogenesis has been largely limited to comparisons of peripheral blood cells^14, 15, 38^. Using the same platform used in this study, 74 methylation sites (29 associated with either the MHC or the human leukocyte antigen (HLA-DRB1 region) were identified in a comparison between CD4+ T cells from patients with MS and healthy individuals^39^. In addition, significant differences in the methylation patterns associated with CD4 and CD8 T cells were detected in MS patients compared to healthy controls^40^. A genome-wide study of methylation in normal appearing white matter from MS patients identified 319 significantly hypermethylated and 220 significantly hypomethylated regions, which mapped to genes involved in oligodendrocyte survival and immune responses^41^. Our study is the first to provide insight into the landscape of methylation changes following demyelination in MS hippocampus. In addition, none of the genes identified in the current study was common to the candidates described in our previous collaborative study using MS normal appearing white matter^41^. This supports the need to develop datasets of gene changes from different gray matter regions in MS brains. Of great interest is the fact that the identified genes are expressed by different cell types in human and mouse brain. While similar DNA methylation studies in mouse are not currently possible due to the lack of a global mouse methylation profile chip, our species-specific cell identity provides the option to query, validate, and manipulate candidate genes for their possible role in memory function using animal models. Future tissue-, region-, and cell-specific analyses of epigenetic changes in MS brains need to be conducted in order to gain further insight into the pathogenesis of MS.

## Materials and Methods

### Human subjects and regulatory compliance

All brains were collected as part of the tissue procurement program approved by the Cleveland Clinic Institutional Review Board. Patient anonymity was strictly maintained and all tissue samples were handled in a coded fashion. All donors or their surrogates gave informed consent for their brains to be used for research studies. All experiments were carried out in accordance with the relevant Cleveland Clinic Institutional regulations and guidelines.

### Tissue collection and characterization

Brains were removed according to a rapid autopsy protocol, sliced (1 cm thick), and then either fixed in 4% paraformaldehyde for morphological studies or rapidly frozen for biochemical analysis. Patient demographics are listed in Table 1. All hippocampi were characterized for demyelination by immunostaining using proteolipid protein (PLP) as described previously^5, 20^. Briefly, frozen 30 µm sections were cut for characterization by immunohistochemistry and for assessment of demyelination. This was followed by collection of 3–4 subsequent sections for DNA isolation. No significant differences in disease duration (27.2 yrs vs 23.2 yrs; p = 0.60) or postmortem interval (7.1 hr vs 7.5 hr; p = 0.87) were detected between the myelinated and demyelinated MS patients.

### Methylation profiling

Genomic DNA was isolated from frozen tissue sections corresponding to regions of myelinated (n = 8) or demyelinated MS hippocampus (n = 7). Genomic DNA was isolated using a QIAamp DNA mini kit (Qiagen Inc, USA) following the manufacturer’s instructions. Purified genomic DNA was processed for bisulfide conversion and subsequent methylation assays. DNA samples were delivered to the Case Western Reserve University Genomics Core Facility, where 1.5 µg of DNA was bisulphite-treated (Zymo EZ DNA methylation gold) per manufacturer’s instructions. Genome-wide methylation profiles were generated using Illumina 450 K methylation arrays. Isolated DNA (100 ng) was used to measure DNA hydroxymethylation with MethylFlash™ Global DNA Hydroxymethylation (5hmC) ELISAs (Epigentek Inc, USA) using a 5hmC mAb-based detection complex.

### Data analysis and cell type identification

Raw data (idat format) were generated through Illumina’s Genome Studio software. Raw data were preprocessed, normalized, and analyses was carried out in the R environment using the ChAMP package, which integrates currently available 450 k analysis methods and also offers its own novel functionality^42^. After running basic quality control metrics, we performed a beta mixture quantile normalization method to adjust for bias introduced by the Infinium type 2 probe design^42^. All DNA methylation data files will be deposited at the GEO (https://www.ncbi.nlm.nih.gov/GEO) and can be accessed through the accession number GSE101658. As part of routine analysis of DNA methylation datasets in our study, the probes on the X chromosome were normalized for males and females separately and independently of autosomal probes because X chromosome inactivation causes significant gender differences in methylation patterns. In addition, to account for age-related DNA methylation changes, we employed the methods described by Horvath *et al*.^43^. Briefly, this multi-tissue predictor of age allows for the estimation of the DNA methylation age of most tissues and cell types. The predictor, which is freely available, was developed using 8,000 samples from 82 Illumina DNA methylation array datasets, encompassing 51 healthy tissues and cell types. Using these methods, we did not detect any differences in DNA methylation for predicted and actual age between MS samples. To determine the cellular identity of the methylation target, identified genes were queried against mouse and human cell-specific RNA sequencing databases^25, 26^. To further validate the results and cellular specificity, we only selected genes that were above the 50^th^ percentile of expression level across all cell types.

### Immunohistochemistry

Sections (30 μm thick) from corresponding (to frozen sections) fixed blocks of the hippocampus were cut on a sliding microtome, microwaved in 10 mM citric acid buffer (pH 6.0) for 5 minutes, incubated in 3% hydrogen peroxide and 1% Triton X-100 in phosphate-buffered saline for 30 minutes, and immunostained by the avidin-biotin complex procedure with diaminobenzidine (DAB) for myelin PLP, MHC Class II, HuR, or other antibodies as described previously^5, 20^. The extent of demyelination and neuronal status were determined from PLP and HuR staining. Using the same protocol, sections were stained using antibodies specific to DNMT1 (1:250; HPA002694; Sigma Aldrich, St. Louis, MO), DNMT3A (1:250; HPA02588; Sigma Aldrich, St. Louis, MO), DNMT3B (1:250; HPA001595; Sigma Aldrich, St. Louis, MO), and α-5-hydroxymethylcytosine (1:500; ab106918; Abcam Inc. Cambridge MA), PLCH1 (1:250; GTX108612; GeneTex Inc. Irvine CA), SFRP1 (1:250; ab94942; Abcam Inc. Cambridge MA).

### Real-time PCR

RT-PCR was performed using a standard TaqMan PCR kit protocol on an Applied Biosystems 7500HT Sequence Detection System using 0.2 μM TaqMan probes. Probes specific to DNMT1 (Hs00154749_m1), DNMT3A Hs01027166_m1) DNMT3B (Hs00171876_m1), TET1 (Hs00286756_m1), TET2 (Hs00325999_m1), TET3 (Hs00379125_m1), PLP1(Hs00166914_m1), AKNA (Hs00363936_m1), WDR81 (Hs00912091_m1), TMEM132B (Hs00287113_m1), EBPL (Hs00831100_s1), HERC6 (Hs00215555_m1), SFRP1 (Hs00610060_m1), NHLH2 (Hs00271585_s1), and PLCH1 (Hs00324566_m1) were used in triplicate reactions. Total RNA was reverse transcribed using a high capacity cDNA reverse transcription kit (Applied Biosystems, Carlsbad, CA). Reverse transcription reactions contained hippocampal RNA, 1X RT random primers (Applied Biosystems, Carlsbad, CA), 0.25 mM dNTP mix, 50 U MultiScripe reverse transcriptase (Applied Biosystems, Carlsbad, CA), and 1 U/μL RNase inhibitor (Applied Biosystems, Carlsbad, CA). TaqMan gene expression assays for multiplex reactions were performed following the manufacturer’s protocol. Each RT-PCR reaction contained 100ng of cDNA product, 1X TaqMan gene expression mix, 100nM GAPDH primers (Applied Biosystems, ID# 4310884E), and 100 nM FAM-labeled target probes for methylated target genes (Applied Biosystems, Carlsbad, CA). Resultant Ct values were normalized and quantitative data are expressed as mean ± S.E.M. The statistical significance of differences between groups in RT-PCR were determined using previously published methods^20^.
